# Continuous imputation of blood gas and metabolic panel laboratory values in the intensive care unit using machine learning

**DOI:** 10.1007/s10877-026-01470-8

**Published:** 2026-07-23

**Authors:** Behrooz Mamandipoor, Martin Krause, Pragnya Korti, Chun-Nan Hsu, Rodney A. Gabriel

**Affiliations:** 1 Division of Perioperative Informatics, Department of Anesthesiology, University of California, San Diego, La Jolla, CA USA; 2 Department of Biomedical Informatics, University of California, San Diego Health, La Jolla, CA USA; 3 Division of Critical Care Medicine, Department of Anesthesiology, University of California, San Diego, La Jolla, CA USA

**Keywords:** deep learning, laboratory imputation, critical care medicine, predictive modeling, artificial intelligence

## Abstract

We developed machine learning (ML) models to perform continuous hourly prediction of arterial blood gas (ABG) and basic metabolic panel (BMP) laboratory values in critically ill patients. We evaluated its impact on prediction of the need for renal replacement therapy (RRT). We compared the performance of the deep learning models using laboratory variables imputed hourly by our ML estimators versus models using laboratory variables imputed by a previous-value baseline. Our ML model incorporated various predictors, including previous laboratory values, administered medications, clinical events, fluid input/output, hemodynamics, and ventilator settings. Accuracy of laboratory imputations were compared using mean absolute error (MAE) and root mean squared error (RMSE). We trained bi-directional long short-term memory models (Bi-LSTM) to predict need for renal replacement therapy (RRT) that differed only in how laboratory variables were imputed over time. We compared performance using area under the receiver operating characteristics curve (AUROC) and area under the precision-recall curve (AUPRC). XGBoost achieved an average error reduction of 33% in MAE and 32% in RMSE across ABG variables, and 19% and 21% across BMP variables, respectively. Bi-LSTM models using our ML-based hourly laboratory estimates (AUROC 0.951, 95% CI 0.942–0.960; AUPRC 0.419, 95% CI 0.375–0.465) outperformed models using previous-value laboratory imputation (AUROC 0.923, 95% CI 0.910–0.935; AUPRC 0.343, 95% CI 0.301–0.386). Our real-time imputation models led to significant error reduction of most ABG and BMP targets, improving deep learning model performance for the need of RRT.

**Supplementary Information:**

The online version contains supplementary material available at https://doi.org/10.1007/s10877-026-01470-8.

## Introduction

Laboratory testing plays a central role in the intensive care unit (ICU), influencing an estimated 70% of diagnostic and therapeutic decisions [1]. In particular, arterial blood gas (ABG) analyses and basic metabolic panel (BMP) measurements are essential for monitoring respiratory function, metabolic status, and electrolyte balance in critically ill patients. These tests guide titration of mechanical ventilation, fluids, and vasoactive drugs, and are often ordered repeatedly over the course of an ICU stay. Despite their clinical value, several studies suggest that up to 50% of routinely ordered laboratory tests have normal results, indicating substantial overuse [1, 2]. Unnecessary laboratory testing increases healthcare costs and exposes patients to avoidable harm, including hospital-acquired anemia, infection risk from repeated phlebotomy or central line access, patient discomfort, and false-positive findings, from hemolysis or blood clumping, that may trigger cascades of additional testing or overtreatment [1–3]. These drawbacks are particularly pronounced for the most commonly drawn laboratory tests, such as ABG and BMP monitoring [4].

Machine learning (ML) offers a promising strategy to mitigate these challenges by providing real-time estimates of laboratory values using routinely collected multimodal ICU data [3, 5]. Accurate ML-based prediction of ABG and BMP variables can provide continuous physiological monitoring while minimizing the number of blood testing needed. Such models have the potential to (i) reduce the frequency of unnecessary laboratory testing, (ii) support earlier recognition of clinical deterioration, and (iii) enable more timely adjustment of ventilation, fluid management, and vasoactive support. Ultimately, real-time prediction of blood test variables could help improve patient outcomes while reducing the cost, invasiveness, and risk associated with frequent laboratory sampling. At the same time, recent advances in deep learning have shown strong performance in modeling complex, high-dimensional ICU data, enabling more precise, dynamic, and individualized risk predictions. However, deep learning models are highly sensitive to the pervasive and often non-random missingness of electronic health record (EHR) data in the ICU. Missing or sparsely sampled laboratory measurements can degrade model performance and lead to unreliable predictions if not handled appropriately. Consequently, robust strategies for estimating or imputing laboratory values over time are essential to support the reliability and clinical utility of deep learning-based prediction models in critical care.

In this study, we developed ML models to perform continuous hourly prediction, using multiple clinical features, of nine ABG and nine BMP laboratory values in critically ill patients. To illustrate a potential clinical application of these estimated laboratory time series and their impact on ICU-based deep learning model performance, we evaluated its impact on early prediction of the need for renal replacement therapy (RRT) occurring anytime during ICU hospitalization after the initial 12 h of ICU admission. We compared the performance of the deep learning models using laboratory variables imputed hourly by our ML estimators versus models using laboratory variables imputed by a previous-value baseline. We hypothesized that our approach to real-time imputation of laboratory values would augment the predictive performance of the deep learning models for RRT prediction.

## Methods

### Study population

The institutional review board at UC San Diego deemed the study exempt from review and participant consent due to the retrospective nature of the study design. We conducted a retrospective cohort study using the Medical Information Mart for Intensive Care IV (MIMIC-IV) database, a large, publicly available, de-identified electronic health record dataset from the Beth Israel Deaconess Medical Center in Boston, Massachusetts [6]. This database encompasses records from more than 90,000 ICU admissions from 2008 to 2022.

### Cohort inclusion and exclusion criteria

ICU stays with a length of stay shorter than 4 h were excluded, as these brief admissions provided insufficient time for meaningful data collection. ICU stays exceeding 30 days were truncated at 30 days - restricting the analysis to the first month of each stay and reducing potential bias from rare, exceptionally prolonged ICU courses. ICU stays were removed if all vital sign measurements—heart rate, respiratory rate, oxygen saturation (SpO₂), and temperature—were completely absent throughout the ICU stay. ICU stays with no recorded ABG or BMP laboratory measurements were excluded from the cohort.

### Outcome definitions for laboratory variable prediction

Our ML models focused on the hourly prediction of ABG and BMP variables. The nine ABG variables included lactate, partial pressure of oxygen (pO₂), partial pressure of carbon dioxide (pCO₂), bicarbonate, base excess, hemoglobin, hematocrit, ionized calcium, and arterial oxygen saturation. The nine BMP variables included creatinine, glucose, blood urea nitrogen (BUN), anion gap, sodium, potassium, chloride, total calcium, and total carbon dioxide. For each target laboratory variable, we trained a separate regression model. At each hour, the outcome was defined as the corresponding laboratory result recorded in the dataset during that hour. Hours without a laboratory measurement for a given variable were treated as missing for that outcome and were not used as labels for supervised model training and evaluation. The objective of the laboratory prediction models was to generate hourly estimates of sparsely observed ABG and BMP variables for continuous trajectory reconstruction and downstream machine learning analyses. These estimates were not intended to replace clinically indicated laboratory measurements.

### Data inputs and preprocessing

To predict hourly ABG and BMP variables, our ML model incorporated various predictors, including two categories of features: time-invariant (static) and time-variant (dynamic) data. Time-invariant variables included age, gender, race/ethnicity, height, weight, body mass index, admission type and location, initial care unit, and insurance status. Time-variant variables included laboratory values, fluid input/ouput, ventilator settings, administered medications, clinical scores (e.g., Glasgow Coma Scale [GCS]), clinical events (e.g., procedures, ), and hemodynamic measurements (e.g., heart rate, respiratory rate, oxygen saturation, blood pressure). Dynamic variables were aggregated into non-overlapping 1-hour windows. Within each window, we computed the mean for continuous variables and the mode for categorical variables when multiple measurements were recorded. The timing of each time-variant variable (such as administered medications) corresponded to the timing of laboratory measurements. Therefore, the model would explicitly capture changes in laboratory values in relation to changes in clinical care. A detailed description of the preprocessing steps for time-variant data is provided in Supplementary Text 1. Procedures for outlier handling and missing-data imputation are described in Supplementary Text 2.

To better capture temporal dynamics, we engineered summary features over recent time windows (12, 24, and 48 h) and over the entire past trajectory up to the current hour for selected high-frequency variables. For each ICU stay and prediction hour, we transformed raw time-stamped clinical data—prior to imputation—into structured hourly features suitable for predictive modeling. Clinical variables were grouped into three frequency categories (high, medium, and low) based on their typical measurement intervals in the training data. For each frequency group, we defined short-term (12-hour), medium-term (24-hour), and long-term (48-hour) retrospective windows. Within each window, we computed measures of central tendency (mean, median, mode), dispersion (standard deviation, median absolute deviation), extremes and range (minimum, maximum, range), and distributional shape (25th and 75th percentiles, and the proportion of observations below or above a median-based threshold). In addition, to characterize patient status over the entire ICU stay up to the current time and capture chronic or persistent abnormalities, we derived trajectory-level features. These included the minimum and maximum values, the proportion of measurements above or below the patient-specific median, and the number of zero crossings relative to a baseline defined by the patient’s average value. All engineered features were computed per stay and per hour using only information available up to, but not after, the prediction time, thereby avoiding any use of future data.

### Data leakage prevention pipeline

A core requirement for clinically trustworthy prediction of laboratory values is strict prevention of data leakage, which can artificially inflate performance estimates and undermine real-world applicability. To minimize this risk, we used a patient-level train–test split, ensuring that no patient contributed data to more than one data split. This design prevents information from the same individual from leaking between training and evaluation cohorts and yields more realistic estimates of out-of-sample performance. For each ABG and BMP variable, we trained a separate model using only those hourly time points where a ground-truth measurement of that variable was available. To prevent temporal and feature leakage at prediction time, we implemented strict censoring of blood-related variables. When predicting a given target variable at hour *t*, we excluded all ABG and BMP measurements (including the target) from hour *t* and from hour *t–1* from the feature set. Models were permitted to use (i) earlier ABG/BMP information from hours ≤ *t–2* and (ii) other available clinical data streams up to hour *t*. This setup is designed to mirror realistic bedside decision-making, where at the time a new laboratory test is being considered, the current hour’s laboratory results and the most recent ABG/BMP measurement may not yet be available. Finally, we avoided using any risk scores or composite indices that directly incorporated the target laboratory value at the prediction hour.The complete list of raw candidate variables used as inputs to the laboratory prediction models is provided inSupplementary Text 3.

### Prediction post-processing and reconstruction of continuous trajectories

We first constrained all model-predicted laboratory values to physiologically plausible ranges specific to each variable. We then reconstructed smoothed laboratory trajectories by combining sparsely observed measurements with model predictions to better characterize laboratory dynamics over time. For each ICU stay and each laboratory variable, we applied a one-dimensional Kalman filter to fuse observed laboratory values and model predictions into a single filtered trajectory. In this dual-measurement framework, observed laboratory values were treated as high-confidence measurements (low measurement variance), whereas model predictions were treated as lower-confidence pseudo-measurements (higher measurement variance). The Kalman filter updated the latent state sequentially over time, producing a smoothed trajectory that remained tightly anchored to true laboratory measurements when available and was guided by model predictions in the intervals between observations.

### Supervised learning of prediction models of laboratory values

We used gradient boosted decision trees (XGBoost) as the primary predictive model for each laboratory variable. A separate XGBoost regression model was trained for each ABG and BMP outcome using the input features described above. As a clinically relevant baseline, we compared our ML predictions against a simple “previous value” strategy. For each hour in the test cohort where a laboratory value was measured, the baseline prediction was defined as the last available measured value of that laboratory (carried forward from prior hours). This baseline mimics a common clinical heuristic of assuming stability in a laboratory value until a new measurement is obtained. To optimize predictive performance, we conducted a randomized hyperparameter search over a predefined space that included the number of trees, maximum tree depth, minimum child weight, learning rate, and tree-level regularization parameters. Models were trained with early stopping based on performance on the validation cohort, using root mean squared error (RMSE) as the primary objective. For each target variable, the best hyperparameter configuration was selected as the one that minimized validation RMSE. After tuning, we refit the model on the training data (with early stopping still guided by the validation data) and evaluate final performance on the held-out test cohort, which was not used for model selection. We evaluated the ML models for laboratory variable prediction using mean absolute error (MAE), RMSE, and the coefficient of determination (R²) on the test cohort. R² was used as a regression performance metric to summarize explained variance and was not interpreted as evidence of clinical interchangeability between predicted and measured laboratory values. For each laboratory variable, we compared the performance of the ML models with the previous-value baseline at hours where ground-truth laboratory measurements were available.

### Renal replacement therapy predictive models

To illustrate a potential clinical application of the ML-estimated laboratory time series and their impact on deep learning model performance, we measured performance improvements for a model predicting RRT initiation (as its usage is highly dependent on trends in laboratory findings). The outcome was the initiation of RRT during the ICU stay at any time after the prediction window (first 12 h of ICU admission). Thus, RRT could be initiated hours to weeks after this prediction window.The observation window was strictly limited to the first 12 h after ICU admission and no laboratory values or clinical variables after the first 12 h were used as model inputs, including during trajectory reconstruction. We restricted the cohort to ICU stays lasting at least 12 h. For each ICU admission, the prediction model took as input the clinical variables measured within the first 12 h and produced a binary label indicating whether the patient would require RRT at any point later during the ICU stay. In MIMIC-IV, RRT-related activities were identified from the “chartevents”, “inputevents”, and “procedureevents” tables using a curated list of item identifiers associated with continuous renal replacement therapy (CRRT) and intermittent hemodialysis (IHD) modalities. An hourly binary indicator of RRT was then assigned based on the presence of these modality-specific item identifiers. Static variables were incorporated into the Bi-LSTM input by encoding them during preprocessing and concatenating them to the dynamic hourly feature vector at each time step. These variables therefore remained constant across the 12-hour input sequence but were available to the sequence model at every time step.

For this downstream clinical prediction tasks, we implemented task-specific deep learning models using bidirectional long short-term memory (Bi-LSTM) neural networks for sequence classification. The Bi-LSTM model took as input the hourly time series of predictor variables over the respective observation window (first 12 h in the ICU). The Bi-LSTM model contained hidden size of 128 units. For each model, outputs from the forward and backward LSTM directions were concatenated and passed to a classification module consisting of a linear layer with 32 units, a ReLU activation, and a dropout layer to reduce overfitting. To improve training stability and convergence, we applied Xavier uniform initialization to all linear and recurrent layers. To address class imbalance, we used weighted random sampling, assigning sampling probabilities inversely proportional to class frequencies to ensure balanced representation of positive and negative cases within training batches. In addition, we employed a weighted cross-entropy loss function that placed greater emphasis on the minority class, helping to maintain balanced training dynamics. Model optimization was performed using the Adam optimizer with a learning rate of 5 × 10⁻⁴ and a weight decay of 0.01. We applied a StepLR learning rate scheduler to reduce the learning rate by a factor of 10 every 20 epochs. Training used a batch size of 16 and was terminated early if no improvement in area under the receiver operating characteristic curve (AUROC) or F1-score was observed over 20 consecutive epochs. A checkpointing mechanism was used to save the model state whenever validation performance improved. The Bi-LSTM architecture and training hyperparameters were selected using random search based on validation-set performance.

To evaluate the impact of laboratory value estimation on downstream clinical prediction, we trained two parallel Bi-LSTM models with identical architecture, hyperparameters, training procedure, cohort splits, outcome definition, and non-laboratory input features. The models differed only in how ABG and BMP variables were represented over time. In the baseline condition, laboratory variables were completed using the previous-value carry-forward strategy. In the ML-based condition, laboratory variables were completed using the hourly estimates generated by the laboratory prediction models. Thus, the downstream comparison was designed to isolate the effect of laboratory trajectory estimation within a fixed Bi-LSTM sequence-modeling framework. We evaluated classifier performance using the AUROC, area under the precision-recall curve (AUPRC), F1-score, accuracy, and Brier score. To quantify statistical uncertainty, we computed 95% confidence intervals for each performance metric using the pivot bootstrap method with 1,000 resamples drawn from the test dataset. AUROCs were compared using DeLong’s test, with the significance level set to 0.01.

For internal evaluation, we partitioned each cohort into training (70%), validation (10%), and test (20%) subsets. To assess temporal generalizability, we performed a temporally-separated external evaluation by training our models on ICU admissions during 2008–2019 (termed pre-COVID-19 era MIMIC-IV cohort) and testing them on patients admitted during the COVID-19 pandemic period from 2020 to 2022 (termed COVID-19 era MIMIC-IV cohort) to assess temporal reproducibility. We considered the pandemic-era cohort to be externally distinct due to substantial shifts in ICU management protocols, resource allocation, and patient characteristics during this period [7].

### Interpretability

To interpret the deep learning models used for RRT risk prediction, and to evaluate the impact of the estimated blood variables on model behavior, we applied the Integrated Gradients (IG) method. IG quantifies the contribution of each input feature to a specific prediction by comparing the model’s output for the observed input with its output for a baseline input that represents the absence of signal (e.g., an all-zero feature sequence of the same length). Positive attributions indicate features that increase the predicted risk for the target class and therefore support the model’s decision, whereas negative attributions indicate features that decrease the predicted risk. For feature-level summaries, signed attributions were first summed across the 12 hourly time steps for each feature within each patient. We then calculated the absolute value of each patient-level summed attribution and averaged these values across patients. Therefore, the reported IG values represent mean absolute attribution magnitudes and were used as relative feature-importance measures. We used these attributions to analyze the relative importance of different clinical variables, including ABG and BMP features, under both the ML-based laboratory estimation and previous-value baseline strategies.

## Results

### Study cohort

In the pre-COVID-19 era MIMIC-IV cohort, 65,707 ICU stays were included in the analysis for the training, validation, and test sets. The median (quartiles) patient age was 66 years (55–77), 56% of patients were male, and the median (quartiles) ICU length of stay was 2.0 days (1.1–3.7). In-hospital mortality and RRT utilization was 6.7%, and 6.0% of ICU stays, respectively. In the COVID-19 era MIMIC-IV cohort, 9,124 ICU stays were included. The median (quartiles) age was 66 years (55–76), 59% of patients were male, and the median (quartiles) ICU length of stay was 2.3 days (1.3–4.7). In-hospital mortality and RRT utilization was 9.3%, and 7.2% of ICU stays, respectively received RRT during the ICU admission. Baseline demographic and clinical characteristics for both cohorts are summarized in Table 1.

**Table 1:** Demographics and data distribution of MIMIC-IV datasets used in the study. Pre-Covid-19 era MIMIC-IV cohort corresponds to all patients prior to 2020, whereas Covid-19 era MIMIC-IV cohort consists of the portion of the MIMIC datasets during the pandemic (2020–2022)

	Total Pre-COVID-19 era MIMIC-IV dataset (training, validation, and test set)	Pre-COVID-19 era MIMIC-IV cohort (≤ 2019) (test set)	COVID-19 era cohort MIMIC-IV cohort (2020–2022)
Total ICU stays (*n*)	65,707	13,144	9124
Age (years), median [quartiles]	66 [55–77]	66 [55–78]	66 [55–76]
Male sex, n (%)	36,736 (55.9)	7407 (56.4)	5371 (58.9)
Race, n (%)			
Asian	1938 (2.9)	381 (2.9)	322 (3.5)
African American	7087 (10.8)	1410 (10.7)	611 (6.7)
Hispanic	2548 (3.9)	539 (4.1)	293 (3.2)
Other	9250 (14.1)	1884 (14.3)	2578 (28.3)
White	44,884 (68.3)	8930 (67.9)	5320 (58.3)
Hispanic ethnicity, n (%)	2548 (3.9)	539 (4.1)	293 (3.2)
Renal replacement therapy needed, n (%)	3912 (6.0)	798 (6.1)	661 (7.2)
ICU length of stay (days), median [quartiles]	2.0 [1.1–3.7]	1.9 [1.1–3.7]	2.3 [1.3–4.7]

Abbreviations: MIMIC, Medical Information Mart for Intensive Care; LoS, length of stay

### Patterns of laboratory measurements

Across laboratory targets, the median (quartiles) number of measurements per ICU stay was 3.0 [1.0–4.0] in the pre-COVID-19 era MIMIC-IV cohort and 4.0 [2.0–4.0] in the COVID-19 era MIMIC-IV cohort. Across variables, the median (quartiles) time between consecutive measurements was 8.0 h [4.0–11.0] in the pre-COVID-19 era MIMIC-IV cohort and 7.0 h [4.0–11.0] in the COVID-19 era MIMIC-IV cohort. As a representative example, lactate was measured 187,848 times in the pre-COVID-19 era MIMIC-IV cohort, corresponding to a mean of 2.86 measurements per ICU stay. Lactate values were observed in 3.58% of ICU hours (96.42% missing), with a median (quartiles) interval of 5 h [3–10] between consecutive measurements. Distributions, sampling density, and timing statistics for all variables are reported in Supplementary Table 1.

### Performance of laboratory value prediction

**Fig. 1:**
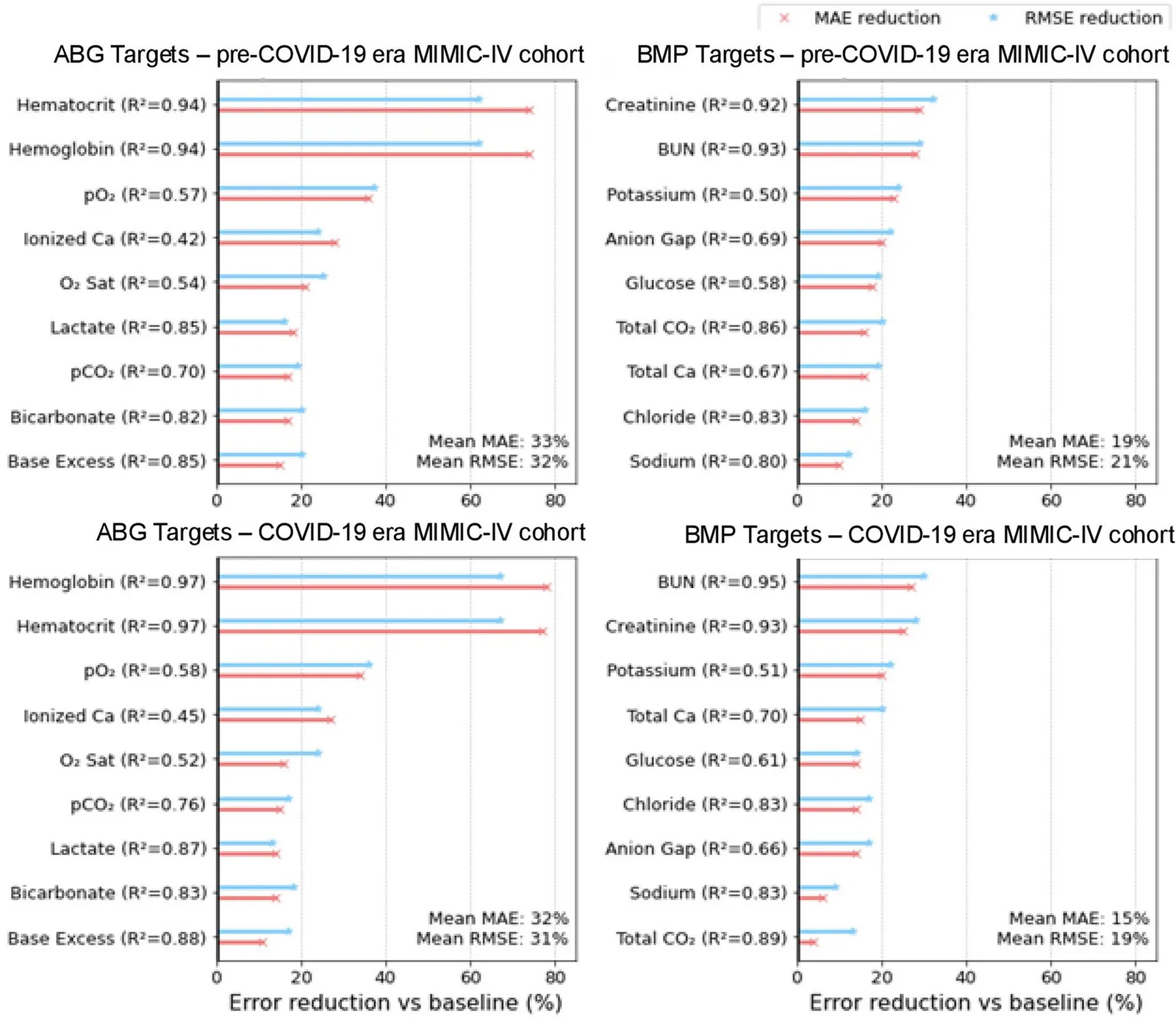
Error reduction for laboratory value prediction across two cohorts. Percent reduction in prediction error relative to a previous-value baseline for arterial blood gas (ABG) and basic metabolic panel (BMP) targets in the pre-COVID-19 era MIMIC-IV cohort (Panels A–B) and the COVID-19 era MIMIC-IV cohort (Panels C–D). For each analyte, filled markers indicate MAE reduction and open markers indicate RMSE reduction (higher values denote larger improvement). Variables are ordered within each panel by MAE reduction to highlight the most improved targets. Across both cohorts, the model reduced error for all targets, with the largest gains concentrated in a subset of variables (e.g., hemoglobin/hematocrit for ABG; BUN/creatinine for BMP), while several targets remained comparatively harder to predict despite consistent improvement. R² denotes the coefficient of determination between XGBoost-predicted and observed laboratory values at measured hours in the test cohorts

We evaluated XGBoost regression models for all ABG and BMP targets on held-out test sets in both the pre-COVID-19 era MIMIC-IV cohort and the COVID-19 era MIMIC-IV cohort, using the previous-value baseline as a comparator. Across all laboratory targets, XGBoost consistently outperformed the baseline in both cohorts. In the pre-COVID-19 era MIMIC-IV, XGBoost achieved an average error reduction of 33% in MAE and 32% in RMSE across ABG variables, and 19% (MAE) and 21% (RMSE) across BMP variables. Similar performance patterns were observed in the COVID-19 era MIMIC-IV, with average reductions of 32% in MAE and 31% in RMSE for ABG variables and 15% (MAE) and 19% (RMSE) for BMP variables. Figure 1 summarizes the percent error reduction for each laboratory target in both cohorts. Supplementary Tables 2 and 3 provides more details on the MAE and RMSE for each individual laboratory value in both cohorts.

### Smoothed and filtered laboratory trajectories

**Fig. 2:**
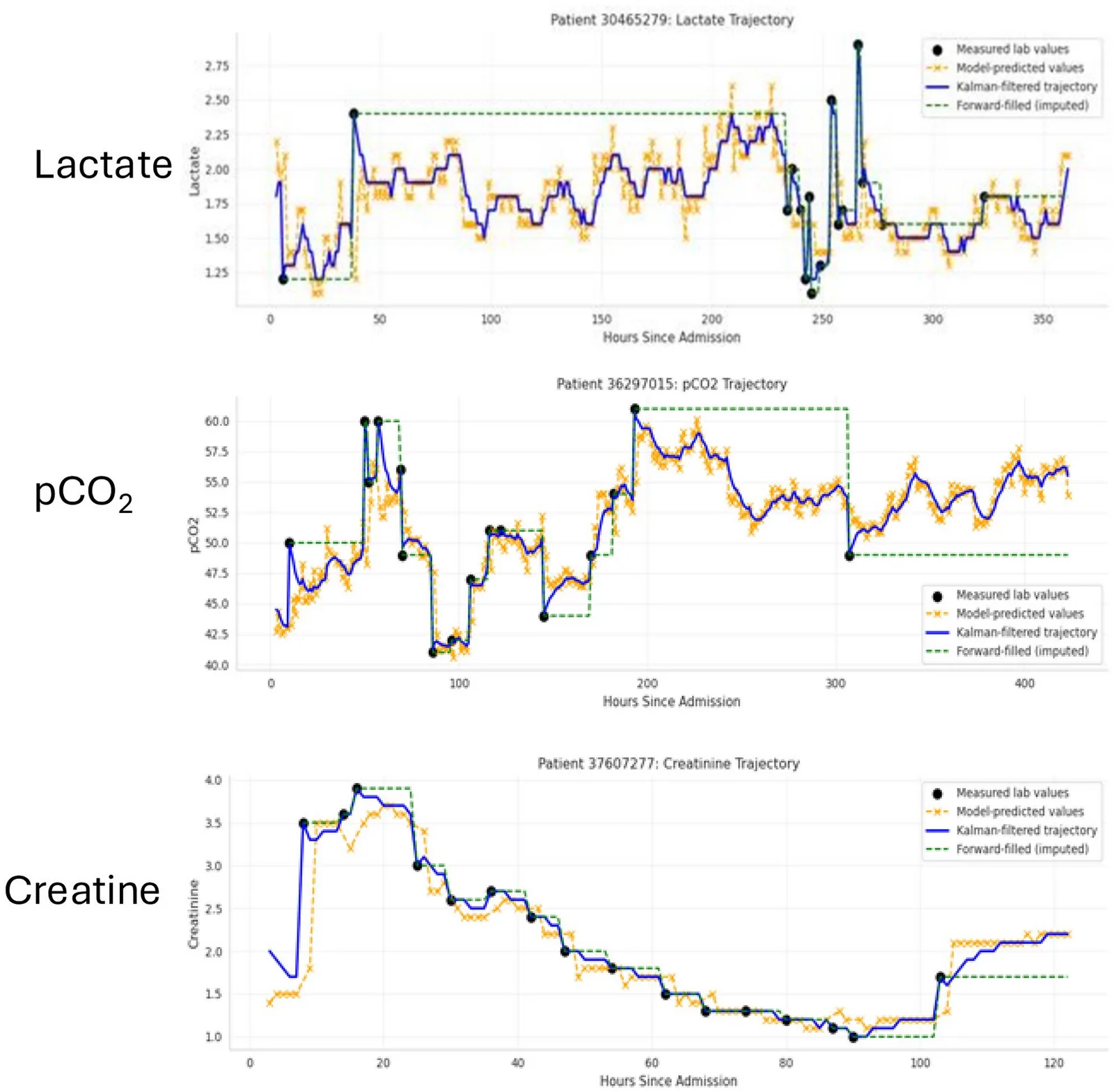
Representative reconstructed laboratory trajectories in selected ICU stays. Time-series examples for lactate, pCO₂, and creatinine illustrate reconstruction of continuous laboratory trajectories by fusing observed measurements with XGBoost-predicted values using a one-dimensional Kalman filter. The Kalman-filtered trajectories closely follow observed values when available and provide smooth, physiologically plausible interpolation during intervals without measurements, where model predictions are incorporated as low-confidence pseudo-measurements. In contrast, the previous-value baseline produces stepwise, piecewise-constant trajectories consistent with last-observation carry-forward behavior

We reconstructed continuous laboratory trajectories by fusing observed laboratory measurements with our ML-generated hourly estimates using a one-dimensional Kalman filter. Figure 2 shows representative examples of lactate, pCO₂, and creatinine trajectories for selected ICU stays. Across examples, the filtered trajectories closely followed true laboratory measurements when available and produced smooth transitions during intervals of sparse sampling. In contrast, the previous-value baseline produced stepwise, piecewise-constant trajectories that reflected last-observation-carry-forward behavior (forward-filled) and did not capture evolving temporal patterns between consecutive laboratory draws.

### Impact of imputed laboratory values on predictive modeling

We next assessed whether improved hourly laboratory estimation translated into better performance on downstream clinical prediction tasks. The use case described here was early prediction of RRT initiation after the initial 12 h of ICU admission (occurrence of RRT anytime during ICU stay after the initial 12 h in the ICU).

**Fig. 3:**
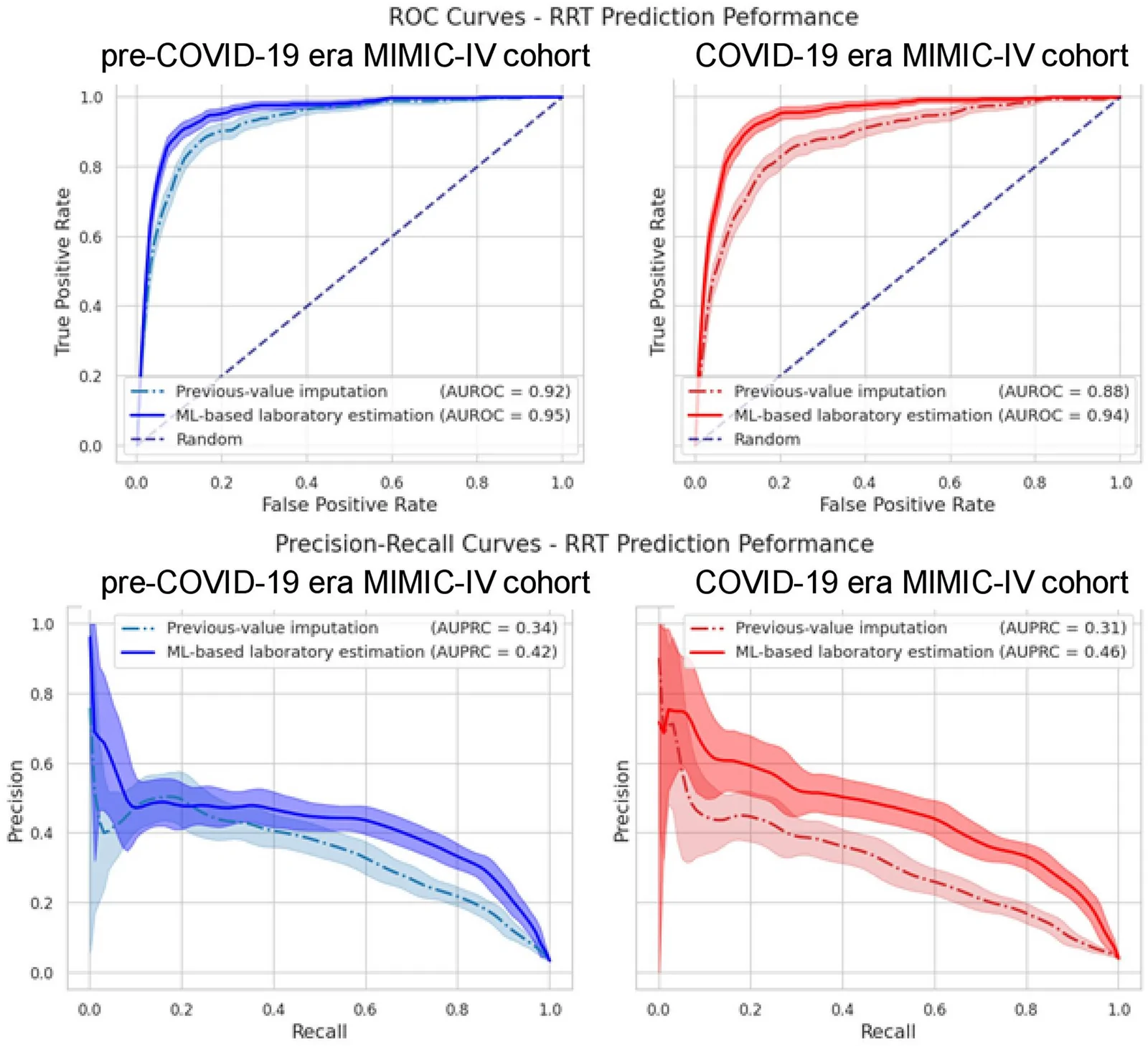
Discrimination performance for renal replacement therapy (RRT) initiation prediction. Receiver operating characteristic (ROC) curves and precision–recall (PR) curves for the Bi-LSTM model predicting subsequent RRT initiation after the initial 12 h of ICU admission, evaluated on the held-out test sets in the pre-COVID-19 era MIMIC-IV cohort and the COVID-19 era MIMIC-IV cohort. Curves compare models using previous-value (last observation carried forward) laboratory imputation (Before) versus machine learning-based hourly laboratory estimates imputation (After). Curves compare two laboratory imputation strategies within the same Bi-LSTM framework: previous-value imputation, in which laboratory variables were completed using the last observed value carried forward, and ML-based laboratory estimation, in which laboratory variables were completed using hourly estimates from the laboratory prediction models. Shaded regions indicate 95% confidence intervals

**Fig. 4:**
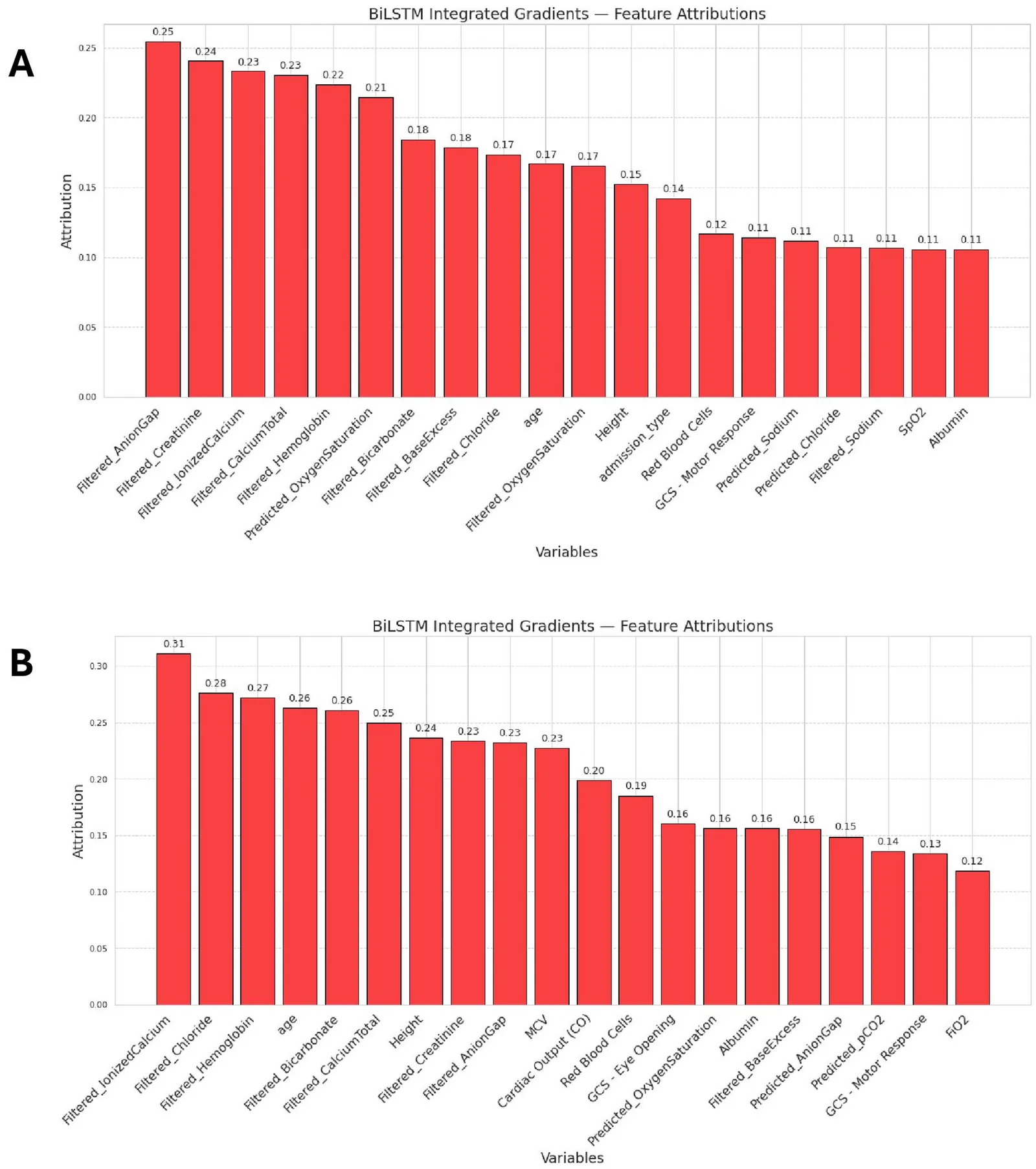
Feature importance for deep learning model predicting need for renal replacement therapy using Integrated Gradients

We excluded ICU stays in which RRT was initiated within the first 12 h of ICU admission. This removed 1,759 stays (3.4%) from the pre-COVID-19 era MIMIC-IV cohort and 289 stays (4.1%) from the COVID-19 era MIMIC-IV cohort. The final analytic cohorts comprised 62,804 ICU stays in the pre-COVID-19 era MIMIC-IV cohort, including 2,129 (3.3%) with subsequent RRT initiation, and 8,604 ICU stays in the COVID-19 era MIMIC-IV cohort, including 356 (4.1%) with subsequent RRT initiation. In the pre-COVID-19 era MIMIC-IV cohort, Bi-LSTM models using our ML-based hourly laboratory estimates achieved strong discrimination (AUROC 0.951, 95% CI 0.942–0.960; AUPRC 0.419, 95% CI 0.375–0.465) and outperformed models using previous-value laboratory imputation (AUROC 0.923, 95% CI 0.910–0.935; AUPRC 0.343, 95% CI 0.301–0.386), corresponding to an absolute improvement of 0.029 in AUROC (paired DeLong test, *p* = 7.9 × 10⁻⁸). Similar patterns were observed in the COVID-19 era MIMIC-IV cohort, where ML-based laboratory estimates yielded an AUROC of 0.944 (95% CI 0.933–0.954) and an AUPRC of 0.459 (95% CI 0.404–0.514), compared with an AUROC of 0.878 (95% CI 0.859–0.896) and an AUPRC of 0.313 (95% CI 0.262–0.360) using the previous-value baseline (Fig. 3). Full performance metrics are reported in Table 2. Integrated Gradients analysis indicated that several estimated laboratory variables were consistently influential predictors of RRT initiation risk, including anion gap, creatinine, ionized calcium, hemoglobin, chloride, bicarbonate, and base excess (Fig. 4).

**Table 2:** Performance metrics of our deep learning model for predicting need for renal replacement therapy on pre-COVID-19 era MIMIC-IV cohort (prior to 2020) and COVID-19 era MIMIC-IV cohort (2020–2022) data from MIMIC-IV comparing how laboratory variables were imputed over time: one set used our machine learning-based hourly estimates, and the other used values carried forward by the previous-value baseline

	AUROC	AUPRC	Brier score	F1-score	Accuracy
Pre-COVID-19 era MIMIC-IV cohort					
“Carry forward” from previous value imputation	0.923 (0.910–0.935)	0.343 (0.301–0.386)	0.068 (0.065–0.072)	0.341 (0.314–0.366)	0.895 (0.889-0.900)
ML-based imputation	0.951 (0.942–0.960)	0.419 (0.375–0.465)	0.057 (0.054–0.060)	0.472 (0.439–0.503)	0.939 (0.935–0.943)
COVID-19 era MIMIC-IV cohort					
“Carry forward” from previous value imputation	0.878 (0.859–0.896)	0.313 (0.262–0.360)	0.053 (0.050–0.057)	0.279 (0.253–0.306)	0.829 (0.821–0.836)
ML-based imputation	0.944 (0.933–0.954)	0.459 (0.404–0.514)	0.052 (0.049–0.056)	0.473 (0.438–0.507)	0.926 (0.921–0.931)

Abbreviations: AUPRC, area under the precision-recall curve; AUROC, area under the receiving operating characteristics curve

## Discussion

Our ML imputation models showed improved error reductions across all 18 ABG and BMP targets compared with models using previous-value baseline imputation. The largest performance gains were observed for hemoglobin and hematocrit values among the ABG targets and for creatinine and blood urea nitrogen values among the BMP targets. Calcium for ABG targets and total carbon dioxide for BMP targets showed only minimal improvement. With our 18 distinct ML imputation models, we improved a deep learning model’s performance compared to previous-value laboratory imputations in predicting the need for RRT after the initial 12 h. To our knowledge, our approach is the first to train a separate ML model for each target laboratory variable. Highly accurate and frequent imputation models provide multiple clinical benefits, such as reducing unnecessary laboratory testing, earlier recognition of clinical deterioration, and enabling more timely interventions while simultaneously improving the interpretability of clinical research.

Missing data in clinical research can lead to bias and, consequently, to incorrect conclusions [8]. Simply dropping data reduces the power of studies and is problematic if missing data is not complete at random. Traditional statistical imputation methods, such as previous-value laboratory, single, or regression imputations, have many disadvantages. Among others, they lack variance, underestimate uncertainty, are prone to skewed data, can break correlations between variables, and might ignore that data are not missing at random. ML models are more robust and can capture more complex patterns but remain vulnerable to the impact of missingness [9]. Separate ML models developed for each laboratory variable, as shown in our study, are a potential solution for more accurate imputations.

Early and accurate prediction of end-organ failure, such as acute kidney injury, pulmonary insufficiency, or hemodynamic instability, is essential for timely interventions in the ICU. Appropriate resource allocation, such as the initiation of RRT, mechanical ventilatory or circulatory support, has shown to improve survival, length of stay, and healthcare costs [10–13]. While deep learning models have shown higher precision in predicting clinical deterioration than traditional scoring systems [14], missingness of data remains a main challenge for accuracy [15]. Our hourly ML-based imputation method significantly improved the accuracy of a deep learning model designed to predict RRT initiation. Given the well-known associations between critical care outcomes and conditions often diagnosed based on laboratory values, such as hypoxia, acidosis, anemia, electrolyte and glucose abnormalities [16–19], our imputation method for laboratory values could allow for faster and more accurate predictions in other DL models predicting end-organ injury, healthcare costs, and quality of life.

Overuse of laboratory tests is well documented in the inpatient setting [1]. More efficient use of laboratory tests can lead to significant cost savings [20] and enhance patient safety by reducing the risk of anemia, bloodstream infections, nerve injury, and false-positive findings [3, 21–24]. Different techniques have been studied to lower the frequency and volume of blood draws, such as avoiding overcollection or utilizing small-volume pediatric tubes, point-of-care blood testing, bundling orders to minimize venipunctures, avoiding the practice of “rainbow labs”, provider education, audits and feedback to providers to compare practice with peers encouraging practicing along guidelines, and Clinical Decision Support tools [25–27]. However, implementation of these interventions requires time-consuming education, ongoing provider compliance, and costly resources. Promising strategies, such as our highly accurate and frequent imputation models for blood gas analyses and metabolic panels, embedded in an institution’s EHR system, might alleviate these challenges.

Our study has several limitations. While most laboratory targets showed significant improvement compared with models utilizing previous-value baseline imputation, calcium and total carbon dioxide showed only minimal improvement, likely secondary to a relatively narrow physiological range and limited short-term variability. Second, our study was limited to BMP and ABG targets, excluding other commonly used laboratory tests, such as coagulation and blood count studies. The development of additional imputation models is, therefore, needed. Third, prediction models do not rely solely on laboratory findings but also on other variables, such as demographics, comorbidities, and imaging findings, which can be missing in large datasets. Fourth, our imputation models were trained on the MIMIC-IV cohort and therefore require external validation on datasets with differing patient demographic and socioeconomic compositions. Finally, the purpose of the proposed approach in this study was to improve continuous representation of sparsely measured laboratory variables for computational modeling and downstream risk prediction. Formal agreement analyses and prospective clinical validation would be required before considering any use of such estimates as substitutes for measured laboratory tests.

To conclude, our real-time imputation models led to significant error reduction of most ABG and BMP targets, improving deep learning model performance for the need of RRT. Our hourly imputation method offers a potential mechanism for faster and more accurate prediction. Further external validation across different datasets is needed to confirm the validity of our imputation models.

## Supplementary Information

Below is the link to the electronic supplementary material.

Supplementary Material 1

## Data Availability

Data is available from MIMIC-IV public repository from PhysioNet.org.
