# Supplementary material for: Continuous imputation of blood gas and metabolic panel laboratory values in the intensive care unit using machine learning
Source: J Clin Monit Comput. 2026 Jul 23;40(5):1091–102. doi: 10.1007/s10877-026-01470-8 (PMC13562130; doi:10.1007/s10877-026-01470-8)
Supplement: Supplementary file 1 [file 10877_2026_1470_MOESM1_ESM.pdf]

Supplementary Text 1

Data pre-processing for time-variant features

For dynamic data (time-variant features), the time-variant features included laboratory values, medications, ventilator settings, clinical scores (e.g., clinical risk scores, Glasgow Coma Scale [GCS], etc.), clinical events (e.g., procedures), and hemodynamics. Categorical variables were numerically encoded, and inconsistencies in variable types were corrected (e.g., converting misclassified strings to floats). Variable names and units were standardized across tables to ensure consistency and comparability. Dynamic variables were binned into non-overlapping 1-hour windows, within which the mean for continuous variables or the mode for categorical variables was calculated when multiple values were recorded within a bin. Hours without any recorded information for a given variable were treated as missing for that variable. This approach allowed us to accommodate varying sampling frequencies across measurements. To standardize urine output measurements in clinical datasets and to support accurate fluid balance assessments, we created a list that included various descriptors and terms used to denote urine output within the dataset. This list encompassed a range of expressions, from general terms like “Urine” and “URINE CATHETER” to more specific descriptors such as “Output External Urethral Device Condom Catheter” and “Urine Output-Foley.” We also calculated three widely recognized clinical severity scores and mortality estimation systems hourly by utilizing the EHR data. These include the PaO_2/FiO_2 ratio, Shock Index, and Oxford Acute Severity of Illness Score (OASIS).

For medication pre-processing, we created extensive lists of drug names for each critical care medication to ensure thorough coverage during the extraction process. We then converted the presence of drugs into a binary representation indicating whether patients received them or not. Vasopressor use was defined based on whether a patient received any of the following medications: epinephrine, vasopressin, milrinone, dobutamine, phenylephrine, or norepinephrine. To identify antibiotic medications, we created a list of known antibiotic substances, including both common names and specific drug identifiers (GSN codes). Any drug name that contained any part of the antibiotic substrings or matched any GSN code was flagged with a binary indicator denoting antibiotic exposure.

Supplementary Text 2

Outlier removal

To ensure data quality and consistency, we first addressed potential entry errors in static features. In particular, patient height and weight values, which were often entered manually, were occasionally swapped. When such inconsistencies were detected (e.g., implausible combinations), the values were corrected by swapping height and weight. For dynamic clinical variables, we applied outlier detection and removal using clinically validated ranges based on medical knowledge specific to each variable. Measurements falling outside these physiologically plausible ranges were treated as outliers and excluded from the analysis.

Missing data imputation

We addressed missing data through a clinically informed imputation approach. Initially, we imputed missing values by leveraging established clinical relationships among variables. For instance, missing Glasgow Coma Scale (GCS) total scores were derived either from available component scores or mapped directly from corresponding Richmond Agitation-Sedation Scale (RASS) scores (e.g., RASS = 0 was mapped to a GCS of 15). Hemoglobin and hematocrit values were imputed based on their known linear relationship. Similarly, bilirubin measurements (total or direct) were imputed using their typical clinical ratio when one measurement was absent. Missing arterial blood pressure values (systolic, diastolic, and mean arterial pressure) were estimated using their known physiological relationships. Additionally, we introduced a binary indicator variable at each time point, indicating whether a given measurement was originally recorded or imputed. We implemented forward-filling of data for predefined intervals based on clinical judgment: up to 24 hours for medication-related variables and up to 12 hours for other dynamic physiological variables. These intervals represented clinically valid active periods during which previously recorded measurements could reasonably reflect the patient's current physiological status. Beyond these intervals—or if no prior measurements existed—we imputed missing values using summary statistics derived exclusively from the training dataset (mean for continuous variables, mode for categorical variables, and zero for binary variables) to prevent data leakage.

Two different imputation procedures were used in this study and were kept separate. First, conventional imputation was used only for preprocessing model input features as described above. This included forward filling of observed values within clinically defined windows and, when no prior value was available, imputation using summary statistics estimated from the training cohort. Second, ML-based continuous laboratory imputation was performed after the XGBoost models were trained. These model-generated hourly estimates were used to reconstruct continuous laboratory trajectories and to compare downstream RRT prediction performance. Kalman filtering was applied only after XGBoost prediction, as a trajectory-smoothing step that fused observed laboratory values with model estimates. Neither XGBoost-generated estimates nor Kalman-filtered values were fed back into the XGBoost models as predictors.

Supplementary Text 3

Variables used for prediction models.
Static variables: hour since ICU admission, weight, height, age, gender, race, admission type, first care unit, insurance status, admission location. Dynamic variables: heart rate, SpO_2, SvO_2, respiratory rate, temperature, blood pressure (mean, systolic, and diastolic); bilirubin, total bilirubin, PT, PTT, INR, AST, ALT, WBC, RBC, RDW, white blood cells, red blood cells, platelet count, MCV, MCH, MCHC, magnesium, phosphate, alkaline phosphate, pH, non-ionized calcium, protein, total protein, total cholesterol, differential bands, differential monocytes, differential lymphocytes, differential eosinophils, differential basophils, differential neutrophils, oxygen, O_2 flow, flow rate, mean airway pressure, plateau pressure, central venous pressure, amylase, albumin, fibrinogen, triglyceride, transferrin, ferritin, troponin T, vancomycin, C-reactive protein, FiO_2, PEEP, tidal volume, ventilation rate, ventilation status, creatine kinase, CK-MB, CK-MB index, cardiac output, total GCS, GCS eye opening, GCS verbal response, GCS motor response, Richmond-RAS Scale, goal Richmond-RAS Scale, pain level, pain present, mental status, risk for falls, delirium assessment, CAM-ICU mental status change, CAM-ICU inattention, CAM-ICU disorganized thinking, CAM-ICU RASS level of consciousness, CAM-ICU altered level of consciousness, Shock Index, OASIS, magnesium non-IV, insulin, potassium IV, propofol, heparin, vasopressor, fentanyl, crystalloid, vancomycin, oral intake, dextrose input/outputs, urine output, norepinephrine, fentanyl, antibiotic, heparin, insulin, propofol, phenylephrine, vasopressin, epinephrine, and pantoprazole exposure.

Feature-generation schema
Dynamic variables were first aggregated into non-overlapping 1-hour bins. For continuous variables, the mean value within each hour was used; for categorical variables, the mode was used. For selected dynamic variables, we generated retrospective summary features using 12-hour, 24-hour, or 48-hour lookback windows based on measurement frequency. These features included mean, median, mode, standard deviation, median absolute deviation, minimum, maximum, range, 25th percentile, 75th percentile, proportion of observations below or above a median-based threshold, and zero-crossing count relative to a patient-specific baseline. Longitudinal trajectory-level features, including historical minimum and maximum values, were computed using only data available before the prediction time. For ABG and BMP variables, current-hour and immediately preceding-hour values were censored for every target model; only values and engineered summaries derived from hour t–2 or earlier were eligible for inclusion. This ensured that all model inputs reflected information that would have been available at the bedside at the time of prediction.

Supplementary Table 1. Distributions, sampling density, and timing statistics for all variables in the pre-COVID-19 era MIMIC-IV cohort (patient data ≤ 2019) and COVID-19 era MIMIC-IV cohort (patient data from 2020-2022). ***** Hours with measurements (%) was calculated as the percentage of eligible ICU stay–hour bins in which the corresponding laboratory variable was observed. Specifically, for each variable, this was defined as: 100 × (number of hourly bins with an observed measurement / total number of eligible ICU stay–hour bins). This metric reflects the sparsity of laboratory measurements across the hourly ICU time series.

Pre-COVID-19 era	Total measurements	Value, median [Q1,Q3]	Interval between measurements, median [Q1,Q3]	Measurements per stay, mean	* Hours with measurements, %
**pH**	353281	7.4 [7.3-7.4]	4 [2-8]	5.38	6.73
**pO2**	292773	102 [74-145]	4 [2-7]	4.46	5.58
**pCO2**	292688	40 [35-47]	4 [2-7]	4.45	5.58
**Bicarbonate**	358222	24 [21-27]	12 [7-22]	5.45	6.83
**Base Excess**	292467	0 [-3-2]	4 [2-7]	4.45	5.57
**Oxygen Saturation**	102517	94 [73-97]	4 [2-7]	1.56	1.95
**Lactate**	187848	1.8 [1.2-2.8]	5 [3-10]	2.86	3.58
**Hemoglobin**	401523	9.6 [8.4-11]	9 [5-22]	6.11	7.65
**Hematocrit**	401523	29 [25.6-33.1]	9 [5-22]	6.11	7.65
**Ionized Calcium**	173877	1.1 [1.1-1.2]	6 [3-10]	2.65	3.31
**Sodium**	416691	138 [135-142]	9 [6-15]	6.34	7.94
**Potassium**	463849	4.1 [3.7-4.5]	8 [4-13]	7.06	8.84
**Chloride**	398875	104 [99-108]	10 [6-16]	6.07	7.6
**Total CO2**	292715	25 [22-28.5]	4 [2-7]	4.45	5.58
**Glucose**	1044259	133 [109-169]	4 [1-6]	15.89	19.9
**Creatinine**	359004	1 [0.7-1.8]	12 [7-21]	5.46	6.84

Pre-COVID-19 era	Total measurements	Value, median [Q1,Q3]	Interval between measurements, median [Q1,Q3]	Measurements per stay, mean	* Hours with measurements, %
**pH**	353281	7.4 [7.3-7.4]	4 [2-8]	5.38	6.73
**pO2**	292773	102 [74-145]	4 [2-7]	4.46	5.58
**pCO2**	292688	40 [35-47]	4 [2-7]	4.45	5.58
**Bicarbonate**	358222	24 [21-27]	12 [7-22]	5.45	6.83
**Base Excess**	292467	0 [-3-2]	4 [2-7]	4.45	5.57
**Oxygen Saturation**	102517	94 [73-97]	4 [2-7]	1.56	1.95
**Lactate**	187848	1.8 [1.2-2.8]	5 [3-10]	2.86	3.58
**Hemoglobin**	401523	9.6 [8.4-11]	9 [5-22]	6.11	7.65
**Hematocrit**	401523	29 [25.6-33.1]	9 [5-22]	6.11	7.65
**Ionized Calcium**	173877	1.1 [1.1-1.2]	6 [3-10]	2.65	3.31
**Sodium**	416691	138 [135-142]	9 [6-15]	6.34	7.94
**Potassium**	463849	4.1 [3.7-4.5]	8 [4-13]	7.06	8.84
**BUN**	357481	23 [14-39]	12 [7-21]	5.44	6.81
**Anion Gap**	356076	14 [12-16]	12 [7-22]	5.42	6.79
**Calcium Total**	322759	8.4 [7.9-8.8]	12 [7-23]	4.91	6.15

COVID-19	Total measurements	Value, median [Q1,Q3]	Interval between measurements, median [Q1,Q3]	Measurements per stay, mean	Hours with measurements, %
**pH**	59870	7.4 [7.3-7.4]	4 [2-7]	6.56	6.78
**pO2**	55654	96 [70-134]	4 [2-7]	6.1	6.3
**pCO2**	55555	42 [36-49]	4 [2-7]	6.09	6.29
**Bicarbonate**	62878	23	11	6.89	7.12

		[20-26]	[6-21]		
**Base Excess**	55496	-1<br>[-4-2]	4<br>[2-7]	6.08	6.28
**Oxygen Saturation**	20155	90<br>[64-96]	4<br>[2-7]	2.21	2.28
**Lactate**	36504	1.8<br>[1.2-3]	5<br>[2-8]	4	4.13
**Hemoglobin**	66543	9.4<br>[8.1-11.2]	9<br>[5-23]	7.29	7.54
**Hematocrit**	66543	29<br>[25.2-34.4]	9<br>[5-23]	7.29	7.54
**Ionized Calcium**	30837	1.1<br>[1.1-1.2]	6<br>[3-8]	3.38	3.49
**Sodium**	74550	138<br>[135-142]	9<br>[5-14]	8.17	8.44
**Potassium**	79405	4.1<br>[3.7-4.5]	7<br>[5-13]	8.7	8.99
**Chloride**	66583	103<br>[99-107]	11<br>[6-18]	7.3	7.54
**Total CO2**	55553	24<br>[21-27]	4<br>[2-7]	6.09	6.29
**Glucose**	172751	137<br>[112-174]	4<br>[1-6]	18.93	19.56
**Creatinine**	62529	1<br>[0.7-1.7]	11<br>[7-20]	6.85	7.08
**BUN**	62250	23<br>[14-39]	12<br>[7-20]	6.82	7.05
**Anion Gap**	62695	12<br>[10-15]	11<br>[7-21]	6.87	7.1
**Calcium Total**	57771	8.5<br>[8.1-9]	12<br>[7-22]	6.33	6.54

Supplementary Table 2. Using data from pre-COVID-19 era MIMIC-IV cohort

Pre-COVID-19 era	MAE			RMSE			R2
ABG	Baseline	ML	Changes	Baseline	ML	Changes	ML
Lactate	0.71	0.58	% 18	1.16	0.97	% 16	0.85
pO2	43.5	28.1	% 36	72	45.1	% 37	0.57
pCO2	5.27	4.37	% 17	7.95	6.44	% 19	0.70
Bicarbonate	1.98	1.65	% 17	2.79	2.24	% 20	0.82
Base Excess	1.78	1.51	% 15	2.73	2.18	% 20	0.85
Hemoglobin	0.81	0.21	% 74	1.19	0.46	% 62	0.94
Hematocrit	2.40	0.63	% 74	3.51	1.34	% 62	0.94
O2 Sat	9.63	7.32	% 21	14.38	10.57	% 25	0.54
Ionized Calc	0.07	0.05	% 28	0.11	0.08	% 24	0.42

MIMIC-IV	MAE			RMSE			R2
BMP	Baseline	ML	Changes	Baseline	ML	Changes	ML
Creatinine	0.26	0.19	% 29	0.62	0.42	% 32	0.92
Glucose	30.0	25.0	% 18	46.0	37.5	% 19	0.58
BUN	4.96	3.55	% 28	9.20	6.5	% 29	0.93
Anion Gap	2.17	1.74	% 20	2.95	2.3	% 22	0.69
Sodium	2.23	2.0	% 10	3.17	2.8	% 12	0.80
Potassium	0.41	0.31	% 23	0.60	0.46	% 24	0.50
Chloride	2.43	2.09	% 14	3.52	2.95	% 16	0.83
Total Calc	0.37	0.31	% 16	0.56	0.46	% 19	0.67
Total CO2	2.03	1.7	% 16	3.05	2.44	% 20	0.86

Supplementary Table 3. From the COVID-19 era MIMIC-IV dataset.

COVID-19	MAE			RMSE			R2
ABG	Baseline	ML	Changes	Baseline	ML	Changes	ML
Lactate	0.74	0.64	% 14	1.29	1.13	% 13	0.87
pO2	38.4	25.5	% 34	65.2	42	% 36	0.58
pCO2	4.73	4.03	% 15	7.06	5.88	% 17	0.76
Bicarbonate	1.78	1.53	% 14	2.52	2.05	% 18	0.83
Base Excess	1.60	1.43	% 11	2.46	2.04	% 17	0.88
Hemoglobin	0.77	0.17	% 78	1.17	0.39	% 67	0.97
Hematocrit	2.35	0.54	% 77	3.52	1.18	% 67	0.97
O2 Sat	10.83	9.04	% 16	16.61	12.58	% 24	0.52
Ionized Calc	0.08	0.06	% 27	0.11	0.09	% 24	0.45

COVID-19	MAE			RMSE			R2
BMP	Baseline	ML	Changes	Baseline	ML	Changes	ML
Creatinine	0.23	0.18	% 25	0.52	0.38	% 28	0.93
Glucose	28.0	24.0	% 14	42.0	36.0	% 14	0.61
BUN	4.72	3.44	% 27	8.54	5.94	% 30	0.95
Anion Gap	2.28	1.97	% 14	3.02	2.52	% 17	0.66
Sodium	2.23	2.08	% 6	3.16	2.88	% 9	0.83
Potassium	0.39	0.31	% 20	0.55	0.43	% 22	0.51
Chloride	2.44	2.11	% 14	3.52	2.92	% 17	0.83
Total Calc	0.34	0.29	% 15	0.54	0.43	% 20	0.70
Total CO2	1.54	1.48	% 4	2.38	2.08	% 13	0.89
